# Impact of Intubator’s Training Level on First-Pass Success of Endotracheal Intubation in Acute Care Settings: A Four-Center Retrospective Study

**DOI:** 10.3390/children9070960

**Published:** 2022-06-27

**Authors:** Jung-Heon Kim, Jae-Yun Jung, Joong-Wan Park, Se-Uk Lee, Meong-Hi Son, Jeong-Yong Lee

**Affiliations:** 1 Department of Emergency Medicine, Ajou University School of Medicine, Suwon 16499, Korea; medjh@aumc.ac.kr; 2 Department of Emergency Medicine, Seoul National University Hospital, Seoul 03080, Korea; matewoos@snu.ac.kr (J.-Y.J.); zzibii@snuh.org (J.-W.P.); 3 Department of Emergency Medicine, Samsung Medical Center, Seoul 06351, Korea; seuk.lee@samsung.com; 4 Department of Pediatrics, Samsung Medical Center, Seoul 06351, Korea; meonghi.son@samsung.com; 5 Department of Pediatrics, Asan Medical Center, University of Ulsan College of Medicine, Seoul 05505, Korea

**Keywords:** critical care, clinical competence, hypoxia, intubation, risk factors

## Abstract

(1) Background: First-pass success (FPS) of endotracheal intubation is more challenging in children than in adults. We aimed to identify factors associated with FPS of intubation in acute care settings. (2) Methods: We analyzed data of children aged <10 years who underwent intubation within ≤24 h of arrival at four Korean emergency departments (2016–2019). Variables were compared according to FPS. A logistic regression was performed to quantify the association of factors with FPS. An experienced intubator was defined as a senior resident or a specialist. (3) Results: Of 280 children, 169 (60.4%) had FPS. The children with FPS were older (median age, 23.0 vs. 11.0 months; *p* = 0.018), were less frequently in their infancy (36.1% vs. 50.5%; *p* = 0.017), and were less likely to have respiratory compromise (41.4% vs. 55.0%; *p* = 0.030). The children with FPS tended to be more often intubated by experienced intubators than those without FPS (87.0% vs. 78.4%; *p* = 0.057). Desaturation was rarer in those with FPS. Factors associated with FPS were experienced intubators (aOR, 1.93; 95% CI, 1.01–3.67) and children’s age ≥12 months (1.84; 1.13–3.02). (4) Conclusion: FPS of intubation can be facilitated by deploying or developing clinically competent intubators, particularly for infants, in acute care settings.

## 1. Introduction

Emergency endotracheal intubation is challenging in children, as shown by a lower rate of first-pass success (FPS) than that in adults (51.8–83.2% [1,2,3,4,5] vs. 85.4% [6]). Emergency intubation by itself increases the chances of procedural complications by three times [7]. In addition, an attempt to intubate for longer than 30 s is associated with a 5.7-fold increase in desaturation [8]. This complication develops more frequently in infants prone to hypoxia-induced cardiac arrest. This difficulty may be worsened by intubators’ performance anxiety during resuscitation [9,10]. The procedural risk justifies predicting FPS of intubation at the initial presentation.

In emergency departments (EDs) and intensive care units (ICUs), known factors for FPS of intubation include emergency medicine (EM) as an intubator’s specialty, rapid sequence intubation (RSI), not being infants, and the intubator’s training level (Table 1) [1,2,3,4,5]. The training level has been defined as limited to one or two specialties, such as EM (Table 1) [3,4,5]. The impact of clinical experience has been underestimated, given that 56.3–95.0% of the children were intubated by residents in some studies [1,2,3].

We aimed to identify factors for FPS with an emphasis on the training level. To assess this factor at the intubator level, an experienced intubator was defined as an intubator in post-graduate year (PGY) 4–5 (ie, a senior resident in Korea) or a specialist [1] and it investigated as an independent variable. Of note, the variable was defined regardless of the specialties available in acute care settings.

## 2. Materials and Methods

### 2.1. Study Design and Setting

This retrospective study was a planned secondary analysis of an emergency intubation dataset-based study on the factors for underuse of RSI that was conducted across four Korean academic hospitals (during writing, in press in another journal). The EDs and ICUs are staffed by pediatric EM (PEM) attending physicians and intensivists or on-duty pediatric residents, respectively (Table S1). Since 2014, members of the Korean Society of PEM have increased from 79 to 339 individuals, thereby indicating a greater availability of specialists in EDs in the country.

In the EDs, children were intubated by the attending physicians or on-duty residents (EM or pediatrics) under the supervision of the attendings. From the EDs, some children were rapidly transferred and intubated at the ICUs by the pediatric intensivists or on-duty residents. In the trauma bay, attending surgeons intubated critically injured children. All providers completed a periodic Pediatric Advanced Life Support provider course or in-house hands-on practice of airway management. During the study, none of the centers implemented uniform pediatric protocols for RSI and video laryngoscopy. In failed airway situations, senior providers or anesthesiologists further attempted intubation or used rescue airways.

### 2.2. Study Population

This study included consecutive children younger than 10 years undergoing intubation in the EDs, ICUs, or wards within 24 h of arrival at the four EDs from January 2016 through December 2019. The exclusion criteria were as follows: intubation before arrival, dead on arrival, and limited information on the intubator’s training level. As for repeated visits of a child, we analyzed data from the first visit.

### 2.3. Definitions

FPS was defined as a successful intubation on the first laryngoscopic view [11]. Desaturation, defined as an oxyhemoglobin saturation <90% or a ≥10% decrease during or within 10 min after intubation [3], was a focus among the peri-intubation adverse events [12]. This can be attributed to the clinical relevance of hypoxemia, the most common cause of pediatric cardiac arrest, regardless of the occurrence of immediate or technical events, such as mainstem bronchus intubation. Other definitions are tabulated in Table S2.

### 2.4. Data Collection

We identified episodes of intubation by a cross-reference of a query for the inclusion criteria with billing codes related to intubation, such as “endotracheal tube measuring 5.5 mm”. The episodes were primarily reviewed by each site investigator. Vague episodes were discussed with the chief investigator for a consensus.

Patient-level variables included the age (months) with proportions of infants, sex, overall and critical comorbidities, high acuity [13], trauma, crash airway, pre-intubation physiologic abnormalities [14], and indications for intubation (respiratory compromise, altered mental status, cardiac arrest, and shock). We analyzed the physiologic abnormalities, considering the rarity of study using such variables.

Intubator-level variables included the first intubator’s training level (PGY 1–5 and specialists), their specialties (EM, pediatrics, and others), the use of RSI, and locations of intubation (ED, ICU, and ward). PEM attendings were categorized by their certified boards, EM, or pediatrics because the subspecialty has not yet been accredited in Korea.

FPS was the primary outcome. The secondary outcomes included desaturation, the overall success of intubation, the total number of attempts, ED-success time, rescue airways, cardiac arrest, ventilator days, and in-hospital mortality.

### 2.5. Statistical Analyses

Data are presented as means with standard deviations or medians with interquartile ranges and as numbers and percentages for continuous and categorical variables, respectively. The variables were compared using Student’s *t*-tests, Mann–Whitney U tests, chi-square tests, or Fisher’s exact tests. A logistic regression was conducted to quantify the association of factors with FPS while adjusting for potential confounders. We used variables with *p* < 0.1 and a priori variables, including age ≥12 months, high acuity, intubation for respiratory compromise, RSI, and EM as the intubator’s specialty [1,2,4]. We used IBM SPSS Statistics for Windows, version 25.0 (IBM Corp., Armonk, NY, USA).

## 3. Results

### 3.1. Patient-Level Variables

**Figure 1:**
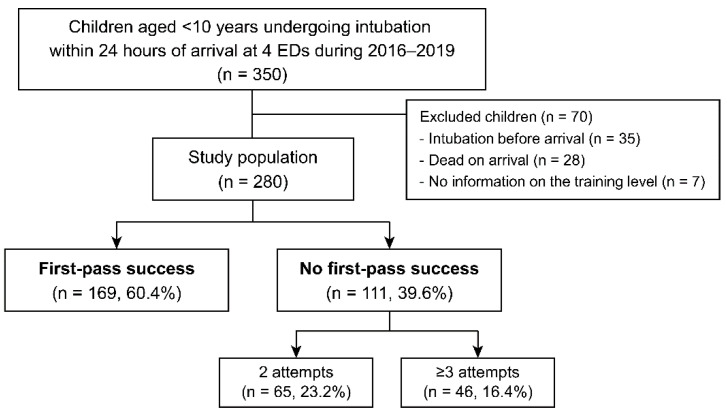
Flowchart for study population. ED indicates emergency department; FPS, first-pass success.

Of 350 eligible children, 280 were included in this study with a 60.4% rate of FPS (Figure 1). The median age was 16.5 months, and 117 children (41.8%) were infants. Of the 111 children (39.6%) without FPS, 46 (16.4%) underwent three or more attempts of intubation. The FPS rates did not differ across the centers (Table S1). Table 2 summarizes the patient-level findings. Children with FPS showed an older median age and lower frequencies of infants and respiratory compromise than the others. The critical comorbidities that hindered intubation are tabulated in Table S3.

No individual pre-intubation physiologic abnormalities differed according to FPS (Table 2). We further analyzed if the presence of experienced intubators masked the potential hindrance of the abnormalities to intubation. As a result, the experienced intubators showed a higher rate of intubating attempts in the high-acuity (experienced intubators, 51.3% vs. non-experienced intubators, 28.3%; *p* = 0.004) or crash airway situations (33.3% vs. 13.0%; *p* = 0.006; Table S4).

### 3.2. Intubator-Level Variables

Children having FPS tended to be intubated more frequently by the experienced intubators, without statistical significances (Table 3). RSI was implemented in 16.4% of the study population. We observed a non-significant increasing tendency of FPS rates with an increase in the training level (20.0%, 51.2%, 60.2%, 69.6%, and 63.7% in the order of each level), experienced intubators (PGY 2–3, 47.8% vs. experienced intubators, 62.8%), and specialists (residents, 58.1% vs. specialists, 63.7%; Table S5). Experienced intubators recorded less frequent desaturation than their counterparts (24.8% vs. 39.1%, *p* = 0.046).

### 3.3. Outcomes

We developed a regression model using the experienced intubators and crash airway, in addition to the five a priori variables. Factors independently associated with FPS of intubation were experienced intubators (adjusted odds ratio, 1.93; 95% confidence interval, 1.01–3.67; *p* = 0.047) and age ≥12 months (1.84; 1.13–3.02; 0.015) (Hosmer–Lemeshow, *p* = 0.947).

Table 4 shows the other outcomes according to FPS. Desaturation was more common in children without FPS (10.1% vs. 53.2%). This group of children had a larger median number of attempts, a longer median ED-success time, and more frequent use of rescue airways than those with FPS. The overall median number of attempts was 1 (range, 1–7). A comparison of the outcomes for three or more attempts displayed a higher frequency of desaturation with a similar pattern of the other comparisons as that for FPS (Table S6).

## 4. Discussion

This study has a two-way implication for endotracheal intubation in acute care settings. First, it is technically difficult to intubate infants who are vulnerable to rapid desaturation, thus highlighting the procedural risk in such situations. Second, experienced intubators who can increase the odds of FPS during intubation by nearly twice may improve the procedural outcomes. This finding confirms the importance of training in upgrading intubation skills, particularly for infants.

Table 1 outlines key findings of the relevant literature. The current study population had a younger median age than the 281 intubated children of a Korean 13 ED study [1]. This difference indicates more difficult intubating conditions in our population [8,15]. The age-related aspect was specified by the low-ranked FPS rate (60.4%) in the 51.8–83.2% reported FPS rates (see median ages, Table 1) [1,2,3,4,5]. The proportion of three or more attempts is parallel to equivalent values ranging from 12.8% to 26.3% [1,3,4]. The frequency of RSI is comparable to the 12.1–25.9% reported in Korea and Japan [1,4] but considerably lower than 80.5% in the United States [2].

The factors for FPS in this study partially differed from the reported factors, such as EM, RSI, and not being infants (Table 1) [1,2,3,4]. Unlike other studies [1,4], EM was not a factor in the current work. This difference might be attributed to the more frequent intubation by pediatricians in our study compared to previous ones (59.9% vs. 15.4–24.9% [1,4]). The bigger role of pediatricians was possibly affected by the participation of two free-standing children’s hospitals (Table S1) and the inclusion of the ICUs (≤24 h of ED arrival). Contrary to the U.S. and Japanese studies [2,4], RSI was not a factor in the current study and another Korean study [1]. This gap may be related to the lower age limits of inclusion in the latter works (<10 years [1] vs. <16–18 years [2,4]) and the inadequate implementation of pediatric RSI in Korea. The diffusion of RSI will facilitate intubation in countries with the protocol being translated to practice. As for “not being infants” as a factor for FPS, it is difficult to intubate infants due to their inherently difficult airways, predisposing them to desaturation [16,17].

We defined the experienced intubators regardless of their specialties to broaden the previous discussion of training level in the limited specialties. A video review on RSI shows the association of an attending physician, defined as a PEM attending or an anesthesiologist, with a 10.2-fold increase in FPS [3]. Another study reported an EM physician in PGY 3 or more as a factor for FPS in children younger than 10 years [4]. In an ICU-based study, pediatric fellows had a stronger association with FPS than did pediatric residents [5]. This current study proves the implications of experienced intubators for pediatric intubation across the specialties available in acute care settings.

In the settings, experienced intubators should be available, supported by the impact of training level on FPS [3,4,5]. Their clinical and psychomotor skills could help overcome a potential hindrance of abnormal vital signs to intubation. In this study, such hindrance was blunted by the experienced intubators’ frequent attempts in crash airway situations, which means combinations of extreme vital signs (Table S4).

Relying on experienced intubators may usurp intubating opportunities by novices, suggesting the need for ongoing training for them. Clinical exposure to intubation may lead to a three-fold increase in procedural confidence [18]. However, pediatric intubation is an infrequent procedure, as demonstrated by the 17.5 intubations per institution per year in this study. Thus, technical competence cannot be achieved and maintained by direct experience alone [17]. A supplementation of the insufficient training warrants a high-fidelity simulation that could enhance novices’ intubating skills [19]. Ideally, the simulation should be repeated every 3–8 months [20,21,22,23]. Considering the association of experienced intubators with FPS, successful intubation can be enhanced by deploying experienced intubators to EDs and ICUs in the short term and by developing clinical competence with ongoing simulation in the long term.

The study has several limitations. First, the study setting may restrict the application of the results to more austere settings. Second, the experienced intubators’ procedural outcomes might be underestimated by the lack of information on individual skill levels, regardless of the training levels, and their frequent involvement in complicated situations (Table S4). Third, unmeasured variables might be confounders. To minimize this potential, we listed the critical comorbidities as a surrogate for difficult airway situations (Table S3). We did not assess the effect of video laryngoscopy, which enhances the glottic view [2,24]. Fourth, the ED-success time might overestimate the temporal delays in some children who did not need intubation in the EDs but only deteriorated later in the ICUs. Finally, some peri-intubation adverse events were probably underreported [3]. This flaw was partially offset by the desaturation, which was available in every record (Table 4).

Briefly, endotracheal intubation, particularly in infants, is inherently challenging, potentially leading to desaturation. This procedural risk can be overcome by deploying or developing clinically competent intubators in acute care settings.

## Figures and Tables

**Table 1:** Literature on factors associated with FPS of intubation in acute care settings.

Author	Study Design/Setting	Factors for FPS ^*^	Training Level	Intubator’s Specialty and Remarks
Choi et al. [1]	Prospective, 13 EDs in Korea, 2006–2010, *n* = 281, age <10 year (median, 23.8 mo; ≤2 year, 50.2%)	FPS, 67.6% EM (2.94; 1.38–6.26)	Not related	EM (72.2%) and PED (24.9%) ≥3 attempts, 12.8%; RSI, 12.1%; and trauma, 28.5%
Kerrey et al. [3]	Video review, single ED in the U.S., Apr 2009–Mar 2010, *n* = 114, age not specified (median, 2.4 year)	FPS, 51.8% Attending (10.20; 2.10–50.90)	Partially related; only 2 specialties (PEM attending or anesthesiologist)	PED residents (42.9%), PEM attendings/fellows (16.1%/18.8%), and EM residents (13.4%) ≥3 attempts, 26.3%; RSI, 100%; and trauma, 18.4%
Goto et al. [4]	Prospective, 17 EDs in Japan, 2010–2014, *n* = 293, age ≤18 year (median, 6 year)	FPS, 60.1% EM (3.21; 1.78–5.83), age ≥10 year (2.45; 1.23–4.87), and RSI (2.17; 1.31–3.57) In <10 year: EM (4.08;1.92–8.63) and RSI (3.05; 1.63–5.70)	Partially related; only 1 specialty (EM in PGY ≥3)	EM (43.3%) and PED (15.4%) ≥3 attempts, 16.0%; RSI, 25.9%; and trauma, 23.5%
Pallin et al. [2]	Prospective, 10 EDs in the U.S., 2002–2012, *n* = 1053, age <16 year (median, 7 year)	FPS, 83.2% Video laryngoscopy (3.40; 1.50–7.60), infants (0.39; 0.26–0.58), and girls (0.57; 0.46–0.73) If excluding “crash airways”: above 3 factors plus RSI (3.40; 1.50–7.40)	Not related	EM (83.6%), PED (6.5%), and PEM (3.6%) ≥3 attempts, unknown; RSI, 80.5%; and trauma, 50.3%
Sanders et al. [5]	Prospective, 15 ICUs in the U.S., 2010–2011, *n* = 1265, age not specified (median, 1 year)	FPS, 60.3% Fellow (4.29; 3.24–5.68; vs. resident)	Partially related; only 1 specialty (pediatrics)	PED (100%) ≥3 attempts, unknown; RSI, unknown; and trauma, 2.4%

* The parenthesized values are adjusted odds ratios with 95% confidence intervals. FPS indicates first-pass success; ED, emergency department; ICU, intensive care unit; EM, emergency medicine; RSI, rapid sequence intubation; PEM, pediatric emergency medicine; PGY, post-graduate year; PED, pediatrics.

**Table 2:** Patient-level variables of the study population.

Variable	Total (*n* = 280)	FPS (*n* = 169)	Non-FPS (*n* = 111)	*p*
Age (month)	16.5 (5.0–52.3)	23.0 (7.0–59.5)	11.0 (4.0–37.0)	0.018
Infants	117 (41.8)	61 (36.1)	56 (50.5)	0.017
Girls	121 (43.2)	73 (43.2)	48 (43.2)	0.994
Overall comorbidity	168 (60.0)	100 (59.2)	68 (61.3)	0.727
Critical comorbidity *	28 (10.0)	14 (8.3)	14 (12.6)	0.238
High acuity	133 (47.5)	86 (50.9)	47 (42.3)	0.161
Trauma	26 (9.3)	17 (10.1)	9 (8.1)	0.582
Crash airway	84 (30.0)	57 (33.7)	27 (24.3)	0.093
Hypotension ^†^	64 (22.9)	38 (22.5)	26 (23.4)	0.855
Tachycardia ^†^	178 (69.8) ^§^	106 (71.1)	72 (67.9)	0.581
Tachypnea ^†^	57 (25.7) ^§^	36 (29.0)	21 (21.4)	0.198
Desaturation ^†^	148 (53.0) ^§^	89 (53.0)	59 (53.2)	0.977
Altered mental status ^†^	110 (39.4) ^§^	69 (40.8)	41 (37.3)	0.553
Indications for intubation				0.030
Respiratory compromise	131 (46.8)	70 (41.4)	61 (55.0)	
Altered mental status	76 (27.1)	47 (27.8)	29 (26.1)	
Cardiac arrest	44 (15.7)	28 (16.6)	16 (14.4)	
Shock or others ^‡^	29 (10.4)	24 (14.2)	5 (4.5)	

The values are expressed as medians (interquartile ranges) or numbers (%). * Refer to the definitions and details in Tables S2 and S3, respectively. ^†^ Refer to the definition of the pre-intubation physiologic abnormalities in Table S2 [14]. ^‡^ Only one 13-month-old febrile boy with pneumomediastinum was intubated at an intensive care unit to prepare endoscopy and later demonstrated a laryngeal injury. ^§^ The denominators are 255, 222, 279, and 279 in the order of rows. FPS indicates first-pass success.

**Table 3:** Intubator-level variables.

Variable	Total (*n* = 280)	FPS (*n* = 169)	Non-FPS (*n* = 111)	*p*
Experienced intubators	234 (83.6)	147 (87.0)	87 (78.4)	0.057
Specialty of intubators				0.166
Emergency medicine	92 (33.0) *	62 (36.9) *	30 (27.0)	0.086 ^§^
Pediatrics	167 (59.9) *	93 (55.4) *	74 (66.7)	
Others	20 (7.2) *^,†^	13 (7.7) *^,†^	7 (6.3)	
RSI	46 (16.4)	26 (15.4)	20 (18.0)	0.561
Induction agents	149 (53.2)	81 (47.9)	68 (61.3)	0.029
Etomidate	21 (7.5)	15 (8.9)	6 (5.4)	0.002
Ketamine	22 (7.9)	17 (10.1)	5 (4.5)	
Fentanyl	17 (6.1)	5 (3.0)	12 (10.8)	
Benzodiazepine	89 (31.8)	44 (26.0)	45 (40.5)	
None	131 (46.8)	88 (52.1)	43 (38.7)	
NMBAs	57 (20.4)	30 (17.8)	27 (24.3)	0.182
Succinylcholine	15 (5.4)	9 (5.3)	6 (5.4)	NA
Rocuronium	3 (1.1)	1 (0.6)	2 (1.8)	
Vecuronium	39 (13.9)	20 (11.8)	19 (17.1)	
None	223 (79.6)	139 (82.2)	84 (75.7)	
Locations of intubation				NA
Emergency department	200 (71.9) ^‡^	121 (72.5) ^‡^	79 (71.2)	
Intensive care unit	73 (26.3) ^‡^	45 (26.9) ^‡^	28 (25.2)	
Ward	5 (1.8) ^‡^	1 (0.6) ^‡^	4 (3.6)	

The values are expressed as numbers (%). ^*^ The denominators are 279 and 168 in the order of columns. ^†^ All but one (by an anesthesiologist) child were intubated by surgeons. ^‡^ The denominators are 278 and 167 in the order of columns. ^§^ According to the presence of emergency medicine physicians. FPS indicates first-pass success; RSI, rapid sequence intubation; NMBA, neuromuscular blocking agent.

**Table 4:** Outcomes.

Variable	Total (*n* = 280)	FPS (*n* = 169)	Non-FPS (*n* = 111)	*p*
Desaturation	76 (27.1)	17 (10.1)	59 (53.2)	<0.001
Overall success	277 (98.9)	169 (100.0)	108 (97.3)	0.061
Total no. of attempts	1.0 (1.0–2.0)	1.0 (1.0–1.0)	2.0 (2.0–3.0)	<0.001
ED-success time (min)	73.5 (22.0–267.8)	49.0 (18.5–208.5)	94.0 (32.0–313.0)	0.008
Rescue airway	4 (1.4)	0 (0)	4 (3.6)	0.024
Cardiac arrest	48 (17.1)	31 (18.3)	17 (15.3)	0.511
Ventilator days	4.0 (1.0–9.0)	3.0 (1.0–8.0)	4.0 (2.0–11.0)	0.217
In-hospital mortality	50 (17.9)	31 (18.3)	19 (17.1)	0.793

The values are expressed as medians (interquartile ranges) or numbers (%). FPS indicates first-pass success; ED, emergency department.

## Data Availability

The datasets analyzed during the current study are not publicly available due to the Korean act of bioethics and biosafety but are available from the corresponding author on reasonable request.

## Supplementary Materials

The following are available online at https://www.mdpi.com/article/10.3390/children9070960/s1, Table S1: Participating centers, Table S2: Definitions, Table S3: Critical comorbidities (*n* = 28), Table S4: High-acuity, crash airway, and pre-intubation physiologic abnormalities according to the training level, Table S5: First-pass success rates according to the training level, Table S6: Outcomes according to three or more attempts.
