# Supplementary material for: Impact of Intubator’s Training Level on First-Pass Success of Endotracheal Intubation in Acute Care Settings: A Four-Center Retrospective Study
Source: Children (Basel). 2022 Jun 27;9(7):960. doi: 10.3390/children9070960 (PMC9322935; doi:10.3390/children9070960)
Supplement: Supplementary file 1 [file children-09-00960-s001.zip › children-1795432-supplementary.pdf]

Table S1. Participating centers

Institution	Free-standing children center	Free-standing trauma center	Annual pediatric visits to the EDs	Study population, n (%)*	FPS, n (%)†
Seoul National University Hospital, Seoul, Korea	Y	N	20,000	79 (28.2)	53 (67.1)
Asan Medical Center, Seoul, Korea	Y	N	40,000	92 (32.9)	55 (59.8)
Samsung Medical Center, Seoul, Korea	N	N	15,000	36 (12.9)	18 (50.0)
Ajou University Hospital, Suwon, Korea	N	Y	25,000	73 (26.1)	43 (58.9)

*The sums of proportions are not equal to 100% due to rounding.

†There was no difference in FPS rates among the four centers (P = 0.364). The denominators are 79, 92, 36, and 73, in the order of rows.

ED indicates emergency department; FPS, first-pass success.

Table S2. Definitions

Term	Definition
First-pass success	Successful intubation on the first laryngoscopic view
Desaturation	S_PO_2 <90% or ≥10% decrease during or within 10 minutes after intubation
Crash airway	Initial S_PO_2 <70% or uncheckable, "U" of the AVPU scale or cardiac arrest
High acuity	Initial level 1 of the Korean Triage and Acuity Scale level 1–5
Experienced intubator	First intubator in post-graduate year 4–5 or a specialist
Critical comorbidity*	Any disease, anomaly, or injury that potentially hinders intubation via airway distortion or obstruction
Pre-intubation physiologic abnormalities[14]	
Hypotension, mmHg	<2 years, systolic blood pressure <70; 2–9 years and <70 + 2 × age (year)
Tachycardia, beats/minute	<2 years, >150; 2–5 years, >140; and 6–9 years, >120
Tachypnea, beats/minute	<1 year, >60; 1–2 year (s), >50; 3–5 years, >40; and 6–9 years, >30
Pre-intubation desaturation	Desaturation prior to a trial of intubation
Altered mental status	"P or U" of the AVPU scale
Rapid sequence intubation	Administration of induction agents and neuromuscular blocking agents to facilitate intubation
Emergency department-success time, min	Time from an arrival to an emergency department arrival to successful intubation

*Refer to the details in Table S3.

Table S3. Critical comorbidities (n = 28)

Variable	No.
Edward syndrome	5
Subglottic stenosis	3
Tracheoesophageal fistula	3
Zellweger syndrome	3
Miller-Dieker syndrome	2
Pierre-Robin syndrome	2
Wolf-Hirschhorn syndrome	2
CHARGE syndrome	1
Krabbe disease	1
Leopard syndrome	1
Nicolaides-Baraitser syndrome	1
Norrie disease	1
Patau syndrome	1
Pompe disease	1
Tracheomalacia	1
Others	0

Table S4. High acuity, crash airway, and pre-intubation physiologic abnormalities according to the training level

Variable	Experienced intubators (n = 234)	Non-experienced intubators (n = 46)	P
High acuity	120 (51.3)	13 (28.3)	0.004
Crash airway*	78 (33.3)	6 (13.0)	0.006
Hypotension†	56 (23.9)	8 (17.4)	0.334
Tachycardia†	145 (68.7)‡	33 (75.0)§	0.409
Tachypnea†	45 (24.9)‡	12 (29.3)§	0.560
Desaturation†	127 (54.3)‡	21 (46.7)§	0.349
Altered mental status†	90 (38.6)‡	20 (43.5)§	0.538

The values are expressed as numbers (%).

*Refer to the definitions and details in Tables S2 and S3, respectively.

†Refer to the definition of the pre-intubation physiologic abnormalities in Table S2 [14].

‡The denominators are 211, 181, 234, and 233 in the order of rows.

§The denominators are 44, 41, 45, and 46 in the order of rows.

Table S5. First-pass success and desaturation according to the training level

Training level	Variable	2†	3	4*	5*	specialist*	P
Each level	FPS	1/5 (20.0)	21/41 (51.2)	59/98 (60.2)	16/23 (69.6)	72/113 (63.7)	0.186
	Desaturation	3/5 (60.0)	15/41 (36.6)	25/98 (25.5)	6/23 (26.1)	27/113 (23.9)	0.255
PGY 2–3 vs. PGY 4–specialist*	FPS	22/46 (47.8)		147/234 (62.8)			0.057
	Desaturation	18/46 (39.1)		58/234 (24.8)			0.046
PGY 2–4 vs. PGY 5–specialist	FPS	81/144 (56.3)			88/136 (64.7)		0.148
	Desaturation	43/144 (29.9)			33/136 (24.3)		0.293
PGY 2–5 vs. specialist	FPS	97/167 (58.1)				72/113 (63.7)	0.344
	Desaturation	49/167 (29.3)				27/113 (23.9)	0.315

The parenthesized values are expressed as %.

*Collectively, the experienced intubators (Table S2). Among the experienced intubators, specialists (n = 113) more frequently attempted to intubate children with high acuity (74 [65.5%] vs. 46 [38.0%]) or trauma (22 [19.5%] vs. 1 [0.8%]) (all P < 0.001), compared to those in PGY 4–5 (senior residents; n = 121).

†No intubator was used in PGY 1.

FPS indicates first-pass success; PGY, post-graduate year.

Table S6. Outcomes according to three or more attempts

Variable	<3 attempts (n = 234)	≥3 attempts (n = 46)	P
Desaturation	48 (20.5)	28 (60.9)	<0.001
Overall success	232 (99.1)	45 (97.8)	0.418
Total no. of attempts	1.0 (1.0–2.0)	3.0 (3.0–4.0)	<0.001
ED-success time (min)	49.0 (18.5–208.5)	94.0 (32.0–313.0)	0.077
Rescue airway	2 (0.9)	2 (4.3)	0.127
Cardiac arrest	42 (17.9)	6 (13.0)	0.420
Ventilator days	3.0 (1.0–8.0)	4.0 (2.0–11.0)	0.115
In-hospital mortality	45 (19.2)	5 (10.9)	0.176

The values are expressed as medians (interquartile ranges) or numbers (%).
